# Hysteresis and Its Correlation to Ionic Defects in Perovskite Solar Cells

**DOI:** 10.1021/acs.jpclett.3c03146

**Published:** 2024-01-29

**Authors:** Sandhya Tammireddy, Muhammad N. Lintangpradipto, Oscar Telschow, Moritz H. Futscher, Bruno Ehrler, Osman M. Bakr, Yana Vaynzof, Carsten Deibel

**Affiliations:** † Institut für Physik, Technische Universität Chemnitz, 09126 Chemnitz, Germany; ‡ KAUST Catalysis Center (KCC), Division of Physical Sciences and Engineering (PSE), King Abdullah University of Science and Technology, Thuwal 23955-6900, Kingdom of Saudi Arabia; ¶ Chair for Emerging Electronic Technologies, Technical University of Dresden, Nöthnitzer Str. 61, 01187 Dresden, Germany; § Leibniz-Institute for Solid State and Materials Research Dresden, Helmholtzstraße 20, 01069 Dresden, Germany; ∥ Laboratory for Thin Films and Photovoltaics, Empa - Swiss Federal Laboratories for Materials Science and Technology, 8600 Dübendorf, Switzerland; ⊥ Center for Nanophotonics, AMOLF, Science Park 104, 1098 XG Amsterdam, The Netherlands

## Abstract

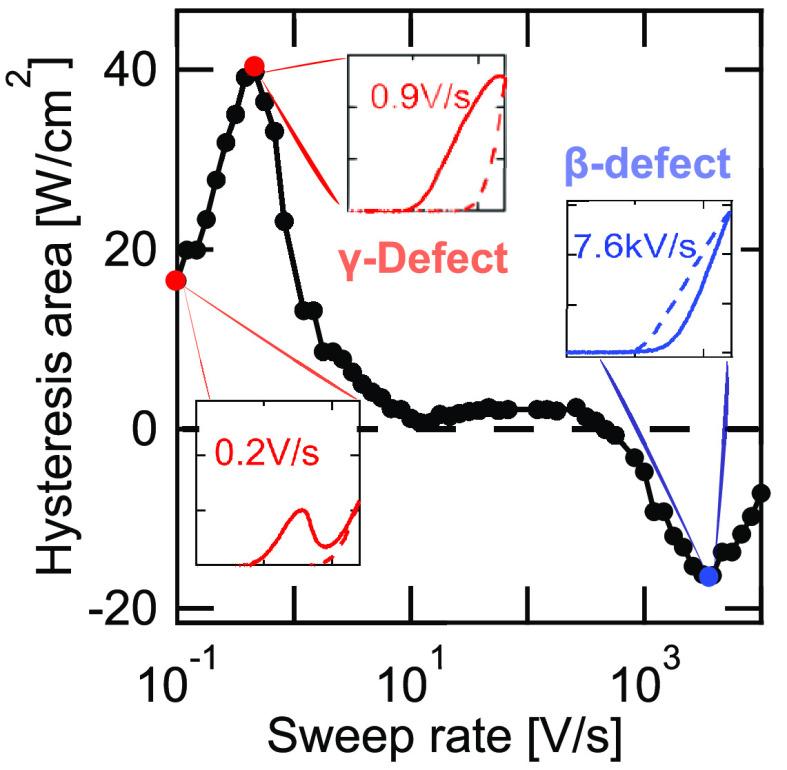

Ion migration has been reported to be one of the main reasons for hysteresis in the current–voltage (*J*–*V*) characteristics of perovskite solar cells. We investigate the interplay between ionic conduction and hysteresis types by studying Cs_0.05_(FA_0.83_MA_0.17_)_0.95_Pb(I_0.9_Br_0.1_)_3_ triple-cation perovskite solar cells through a combination of impedance spectroscopy (IS) and sweep-rate-dependent *J*–*V* curves. By comparing polycrystalline devices to single-crystal MAPbI_3_ devices, we separate two defects, β and γ, both originating from long-range ionic conduction in the bulk. Defect β is associated with a dielectric relaxation, while the migration of γ is influenced by the perovskite/hole transport layer interface. These conduction types are the causes of different types of hysteresis in *J*–*V* curves. The accumulation of ionic defects at the transport layer is the dominant cause for observing tunnel-diode-like characteristics in the *J*–*V* curves. By comparing devices with interface modifications at the electron and hole transport layers, we discuss the species and polarity of involved defects.

Metal halide perovskites have attracted considerable attention as excellent candidates for application in optoelectronic devices, such as solar cells. Despite their excellent performance, photovoltaic devices based on perovskite absorbers may exhibit undesired behavior, such as current–voltage hysteresis. This dynamic and thermally activated effect originates from a delayed current response of the device with respect to a change in the external voltage.^1,2^ Several mechanisms have been proposed as the reasons for hysteresis in perovskites, such as charge carrier trapping and detrapping,^3,4^ ion migration,^5,6^ ferroelectric polarization,^7,8^ and capacitive effects.^9,10^ It is widely acknowledged that the accumulation of defects at the perovskite/transport layer interface causes hysteresis by influencing the electric field distribution within the device. Several theoretical and experimental studies deduced the relevant time scales related to the migration of defect species in perovskites via quantifying hysteresis as a function of temperature and sweep rate of a *J*–*V* measurement.^2,11−14^ However, the origin of the ionic defects is still under debate.

In order to understand the hysteresis in perovskite solar cells, it is essential to locate the ionic defects and estimate their concentration in the device. One particularly powerful method for accessing this information is impedance spectroscopy. In general, reported impedance spectra for perovskite solar cells show both high- and low-frequency responses. The high-frequency response has been attributed to geometric capacitance, chemical capacitance, dipole depolarization, or ionic diffusion in the perovskite active layer.^15−18^ The low-frequency response has been attributed to the impedance of trap states,^19,20^ degradation in the device, dielectric effect,^21^ electron accumulation at the contacts,^22^ and ionic diffusion.^23^ We have reported both low- and high-frequency responses and attributed their origin to ionic defects.^24,25^ These partly contradicting interpretations limit our understanding of defects in perovskites, making it difficult to elucidate their role in affecting device hysteresis. To address this challenge, we combine sweep-rate-dependent *J*–*V* characterization and impedance spectroscopy (IS), which provides a unique opportunity to narrow down contradicting assumptions and allow the direct correlation of defect signatures to hysteretic behavior.

In this work, we report on the conductivity of ionic defects in a series of triple-cation perovskite solar cells Cs_0.05_(FA_0.83_MA_0.17_)_0.95_Pb(I_0.9_Br_0.1_)_3_ with interface modifications using IS and sweep-rate-dependent *J*–*V* curves as a function of temperature. In order to confirm the location of defects, we performed IS on an MAPbI_3_ single-crystal device for reference. We observe two types of defects, β and γ; both originate from the bulk due to the long-range conduction of ionic defects. The conduction of defect β is associated with dielectric relaxation, while the conduction of γ is limited by the perovskite/transport layer interface. The former leads to negative hysteresis, while the latter causes positive hysteresis in the *J*–*V* characteristics of these devices.

## Types of Ion Conduction

In an ionic conductor, it is typical to distinguish between two transport mechanisms of ionic defects. On a microscopic scale, an ionic defect hops between neighboring sites, which is commonly referred to as “short-range conduction”. On the other hand, the classic ionic conductivity is a continuous transport of ionic defects through the lattice, i.e., the “long-range conduction”.^26^ In this case, ion blocking transport layers can be considered as a boundary condition.^27,28^ Impedance spectroscopy allows us to experimentally distinguish these two conduction mechanisms.

Impedance spectroscopy uses an alternating voltage signal with a small perturbation amplitude sweeping across a large range of angular frequencies (ω). By modeling the solar cells as a capacitor and resistor in parallel, *C*∥*R*, the conduction mechanisms involved in the device can be analyzed. In an ideal *C*∥*R* circuit, the characteristic frequency ω_c_ is the inverse of the relaxation time or time constant τ = 1/ω_c_.

By displaying the frequency-dependent data through different mathematical expressions, impedance (Z*), dielectric permittivity (ϵ*), modulus (M*), and loss tangent (tan(ϕ)), the conduction mechanisms can be separated:^29^
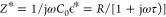
1
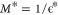
2
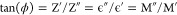
3
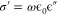
4where *C*_0_ = *A*_0_ϵ_0_/*d* is the geometric capacitance, *d* is the electrode distance, *A*_0_ is the cross-sectional area, and ϵ_0_ is the vacuum permittivity. The superscripts *, ′, and ″ represent complex, real, and imaginary parts, respectively. For a single relaxation process, the corresponding relaxation times in permittivity, loss tangent, impedance, and modulus representations appear in the following order: τ_ϵ_ ≥ τ_tan(ϕ)_ > τ_*Z*_ ≥ τ_*M*_.^30^

At high frequencies, short-range conduction is often expected.^26^ If the spectrum contains short-range ionic conduction, due to variations in the mathematical representation of modulus and impedance, different relaxation times are observed, i.e., τ_*Z*_ ≠ τ_*M*_.^30^

When long-range ionic conduction is present at low frequencies, the imaginary part of permittivity can be written as
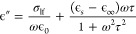
5where ϵ_s_ and ϵ_*∞*_ are the dielectric constants as ω approaches 0 and infinity, respectively. The low-frequency approximation of conductivity σ_lf_ is not identical to steady-state conductivity where the conductivity of ions blocked at the interface would be zero. Because of the dominant long-range ionic conductivity, the permittivity relaxation time does not exhibit a maximum at ω = 1/τ_ϵ_; instead, the spectrum will appear a as straight line with a decreasing slope.^30,31^ However, the reciprocal of the complex permittivity, modulus M* = 1/ϵ*, does show a peak. The relaxation times of modulus and impedance are equal, i.e., τ_*M*_ ≈ τ_*Z*_. A graphical illustration of identifying short-range and long-range conduction is shown in Figure S1.

Another approach to distinguish conduction types is through a fitting of the frequency-dependent conductivity σ(ω, *T*) by the Jonscher universal power law for disordered solids.^32^ The origin of the frequency dependence of the conductivity lies in the relaxation of mobile ions, i.e., short-range conduction. This model predicts upper and lower limits for conductivity and in between a power law behavior. In general, both ionic conductivity and dielectric relaxation processes^33,34^ may be present in a material. Hence, the total frequency- and temperature-dependent conductivity is expressed as
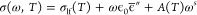
6where *A* and *s* are temperature-dependent parameters and  corresponds to the additional dielectric relaxation. The parameter *s* represents the degree of interaction between mobile ions and their neighbors, and *A* determines the dielectric strength. A value of *s* < 1 indicates ionic conduction leaving the neighborhood, i.e., long-range conduction, while *s* > 1 represents short-range conduction.^35^

Due to the dominant ionic conductivity, the low-frequency dielectric relaxation will be hidden in all the above-mentioned representations. Since the real part of the permittivity ϵ′ or capacitance will not be influenced by σ_lf_, their derivative with respect to frequency, i.e., (*∂ϵ*′/∂ln ω)/ϵ′ shows a peak corresponding to the low-frequency dielectric relaxation.^31^

In general, both long-range and short-range conduction may occur in a given device, i.e., at an interface or/and in the bulk.^30^ Based on the resistive and capacitive contributions, it is possible to locate the defects in the frequency-dependent spectra of IS. Traditionally, since capacitance is inversely proportional to layer thickness, the high capacitance observed at lower frequencies is attributed to thin layers, i.e., interfaces or grain boundaries.^29^ Since the peak heights of *Z*″ and *M*″ are proportional to resistance (*R*) and inverse of capacitance (1/*C*), respectively, a combined representation of the imaginary parts of impedance and modulus makes it possible to distinguish between bulk and interface or grain boundary effects depending on their respective conductivity contribution.

## Results and Discussion

We studied four configurations containing polycrystalline triple-cation solar cells with interface modifications of either the electron transport layer (ETL) or the hole transport layer (HTL) and both. The reference device contains an ITO/PTAA/triple-cation/PCBM/BCP/Ag structure where PTAA (poly [bis(4-phenyl) (2,4,6-trimethylphenyl) amine]) is a hole transport layer and PCBM ([6,6]-phenyl-C60-butyric acid methyl ester) is an electron transport layer. An organic molecule 4-fluoro-phenylethylammonium iodide (F-PEAI) was introduced as a thin layer on top of the hole transport layer (HTL-modified), or before the deposition of the electron transport layer (ETL-modified), or on top of both transport layers (dual-modified). We note that these interfacial modifications do not change the perovskite structure. A brief description of device structures can be found in Methods, while an extensive investigation of device performance, stability, and structural characterization for reproducible samples was described in ref (36).

**Figure 1:**
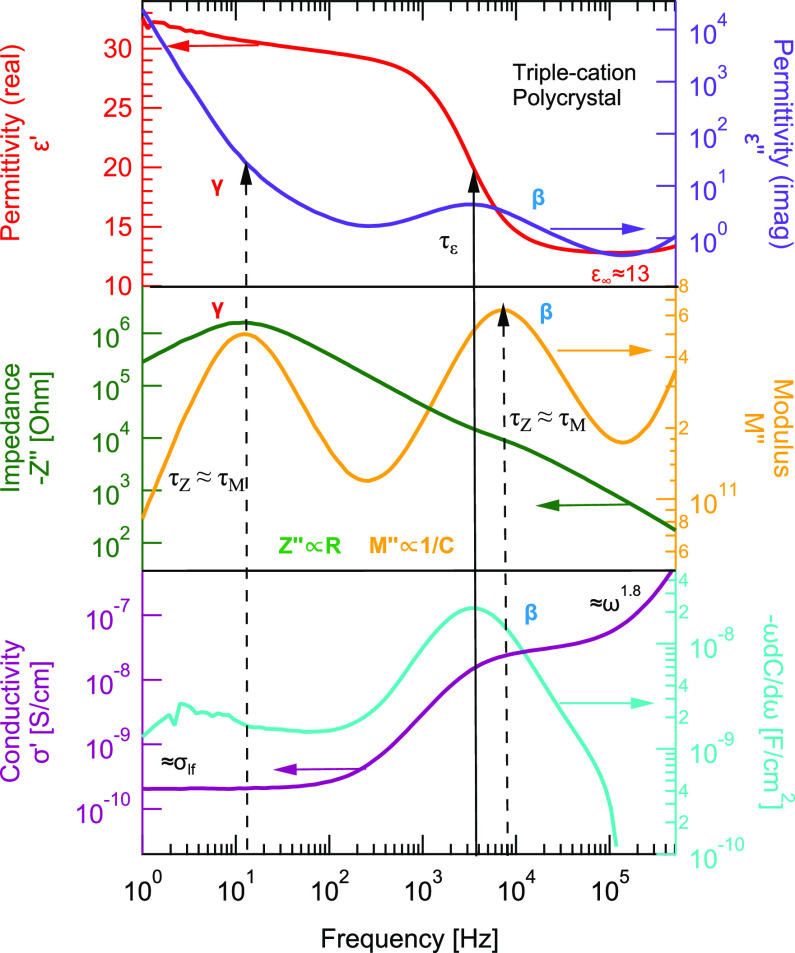
Impedance, permittivity, modulus, and conductivity representations of a triple-cation solar cell measured at 360 K. Two responses β and γ are visible in intermediate and low-frequency regimes, respectively. The relaxation times of γ and β in modulus and impedance representation are equal τ_*M*_ ≈ τ_*Z*_, while τ_ϵ_ does not have finite value for γ. Hence, γ and β correspond to long-range conduction of ionic defects. Since τ_ϵ_ does exist for β, there is dielectric relaxation associated with conduction of β. A power law behavior can be seen at higher frequencies.

The IS measurements were performed under short-circuit and dark conditions. There are two responses in the IS spectrum of all polycrystalline triple-cation devices, a low-frequency response γ (<10^2^ Hz) at high temperatures (>300 K) and a high-frequency response β (>10^2^ Hz), present at all temperatures. An example of impedance, permittivity, and modulus representations along with frequency-dependent conductivity is shown in Figure 1. Based on our previous work, we note that similar impedance spectra were observed in other polycrystalline perovskites such as MAPbI_3_ and CsPbI_3_^25,37^ with comparable activation energies for β and γ, respectively. By comparing the defect signatures observed in deep level transient spectroscopy (DLTS), impedance spectroscopy to intensity modulated photovoltage spectroscopy (IMVS), where transport and recombination signatures can be separated, we found that these two defects are ionic in nature.^24,38^ We further confirmed our approach by comparing conductivities obtained through these methods to literature.^25,39^

In this work, we show that the same defect features are responsible for current–voltage hysteresis, indicating that the electronic current has hysteresis due to the distribution of ionic defects.

**Figure 2:**
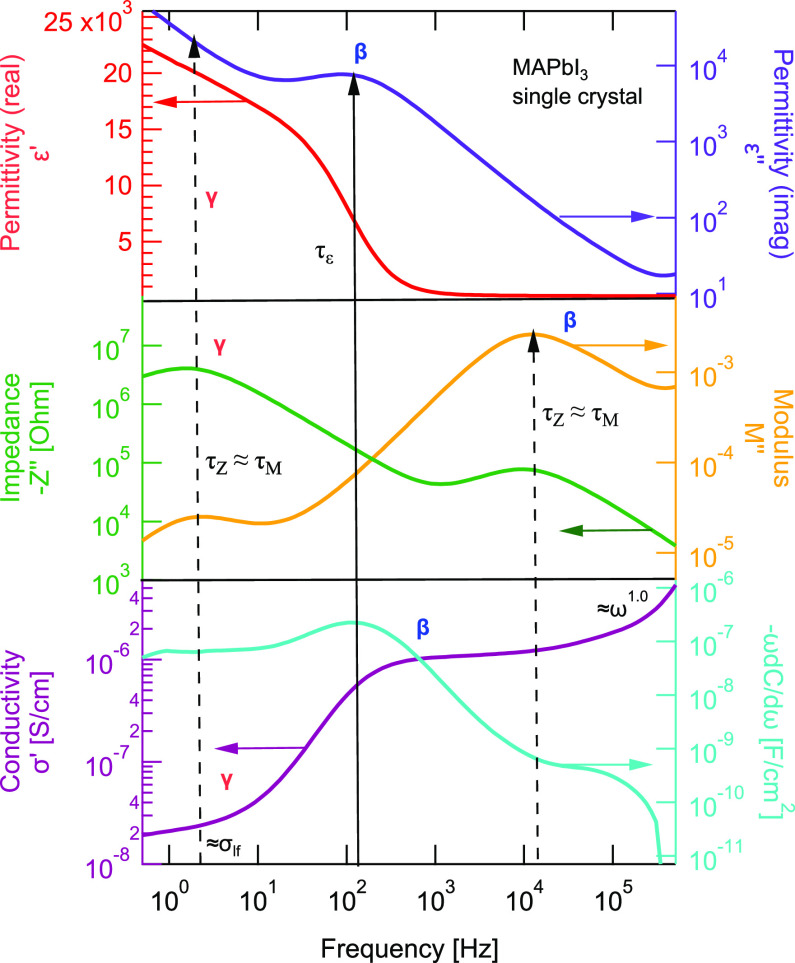
A combined representation of permittivity, modulus, impedance, and conductivity of single-crystal MAPbI_3_ device measured at 360 K. Two defects are observed in the expected range for β and γ originating from ionic conduction. Additionally, β is associated with dielectric relaxation. Two orders of magnitude difference in resistance (*Z*″ ∝ *R*) contribution of γ and β. Hence, β originates from the bulk. Due to absence of grain boundaries, migration of γ is limited by perovskite/transport layer interface.

Since the polycrystalline triple-cation perovskite solar cell consists of bulk, grain boundaries and two transport layer interfaces, it is challenging to locate the defects in the device. In order to simplify the complexity, we investigated a single-crystal MAPbI_3_ solar cell as a reference structure. The single-crystal MAPbI_3_ device is 26 μm thick and shows the expected range of permittivity values.^40^ An example with different representations of the spectra is shown in Figure 2.

We first describe γ and β in terms of their conduction types and locations in the device. Later on we explore their implications in terms of *J*–*V* curve hysteresis through devices with interface modifications.

### Low-Frequency Defect: γ Peak

The real part of the permittivity ϵ′ shows γ as a steady increase with frequency, while the imaginary part of the permittivity indicates a steep rise with a decrease in frequencies for single-crystal MAPbI_3_ device (Figure 2) and triple-cation polycrystalline device (Figure 1). The imaginary parts of the impedance *Z*″ and the modulus *M*″ show γ as distinct peaks with identical relaxation times. This suggests that γ is a bulk response that originates from the long-range conduction of ionic defects in both devices. The low-frequency approximation of conductivity was determined based on the resistance contribution of the defect as
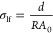
7where *R* corresponds to the maximum resistance caused by defect γ. The activation energies (*E*_A_) can be further deduced from the Arrhenius law as
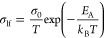
8where σ_0_ is the pre-exponential factor, *k*_B_ is the Boltzmann constant, and *T* is the temperature.^34,41^ Activation energies of 0.47 and 1.08 eV are observed for polycrystalline triple-cation and single-crystal MAPbI_3_ devices, which are consistent with the literature.^42^ The corresponding conductivity σ_lf_ can also be seen at low frequencies (<10 Hz) in the frequency-dependent conductivity plot σ′.

In the polycrystalline triple-cation and single-crystal MAPbI_3_ devices, the major contribution to the impedance *Z*″ originates from γ. Since the peak height is proportional to resistance (*Z*″ ∝ *R*), such a large resistance value indicates that the conduction is occurring at a thin layer such as at an interface,^29^ i.e., a grain boundary or perovskite/transport layer interface. Since there are no grain boundaries in the single-crystal MAPbI_3_ device, this indicates that the migration of the γ defect is limited by one of the perovskite/transport layer interfaces.

A major difference between the single and polycrystalline spectra is the imaginary part of the modulus *M*″. As its height is proportional to 1/*C*, the capacitance contribution of γ in the single-crystal MAPbI_3_ device is larger compared to that of a polycrystalline triple-cation device. We note that this difference is not limited by perovskite type; that is, the polycrystalline MAPbI_3_ device also showed similar behavior. This implies that the electric field created by the Coulomb interaction of ionic defects at the interface is larger^27^ in the single-crystal MAPbI_3_ device as compared to that in the polycrystalline triple-cation device. This difference may change the potential well heights for ion migration, leading to the observation of a different activation energy. In addition, the absence of grain boundaries in single crystals would also give the highest energy barrier for ion migration^42^

Hence, although the activation energies of γ differ in polycrystalline triple-cation and single-crystal MAPbI_3_ devices, they likely have the same origin. We note that a detailed description of such an ionic defect conduction in the presence of blocking contacts was theoretically explained by M. E. Lines through a harmonic oscillator analogy and can be found in refs (27 and 28).

### High-Frequency Defect: β Peak

In the polycrystalline device, the imaginary part of impedance *Z*″ only shows β as a minor shoulder, while the single-crystal device shows a clear β peak. Modulus *M*″ indicates β as a distinct peak in both polycrystalline and single-crystal devices. The relaxation times are comparable in both devices, τ_*Z*_ ≈ τ_*M*_. As explained earlier in the theory section, this can be interpreted as evidence for long-range conduction. However, the real and imaginary parts of the permittivity show β as a distinct step and peak. According to the literature, this suggests that there is a dielectric relaxation involved in the conduction process.^43^

The conductivity above 10^5^ Hz follows a power law behavior with an exponent *s* of 1.8 and 1.0 in polycrystalline triple-cation and single-crystal MAPbI_3_ devices, respectively. The conductivity spectrum of MAPbI_3_ devices was previously studied and shows similar response at low, intermediate, and high-frequency regimes.^44^ An example of the fitting procedure according to eq 6 along with the relevant parameters can be found in the SI. Exponents lower than unity are seen as a sign of long-range conduction of ionic defects. Hence, we conclude that the conductivity in the β and γ regions corresponds to long-range conduction.

For a material in which the dielectric response is only dominated by ionic conduction, no loss peaks are observed.^43^ However, the binding or recombining of ionic defects temporarily with their neighbor would lead to a loss peak in permittivity.^34^ In such a case, the dielectric relaxation depends on the concentration of the ionic defects. In our recent work, we studied MAPbI_3_, triple-cation polycrystalline devices as a function of fractional changes in iodine concentration and observed the same trends in defect densities for β.^25^ This suggests that β corresponds to the dielectric relaxation. Based on the observed trends in densities as well as defect formation, migration enthalpy, and entropy values, we attribute β to a halide, likely an iodine related defect.

In contrast to MAPbI_3_ perovskite, the reported conductivity spectrum of MAHgCl_3_, (C_2_H_5_NH_3_)_2_MnCl_4_ perovskites does not show any dielectric response.^45,46^ This also suggests that lead, iodide, or likely an interaction between both might be the origin of the observed defects.

Analogous to polycrystalline devices, the single-crystal device shows defect β with similar activation energy (0.5 eV). This further confirms that β originates from the bulk of the perovskite in both devices.

### Interpretation of Sweep-Rate-Dependent *J*–*V* Curves

A *J*–*V* curve is a quasidynamic measurement obtained through sweeping the applied voltage within a particular time. A delayed current response leads to time-dependent hysteresis, which can be probed by performing forward and reverse sweeps at different sweep rates
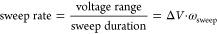
9

Usually the sweep rate is varied by sweeping through different frequencies ω_sweep_, while the voltage range Δ*V* is kept constant. By taking into account that the hysteresis loop area (HA) has a physical meaning, i.e., a measure of power dissipated per sweep due to redistribution of ionic charges, it can be quantified as the difference between the integrated *J*–*V* curves as follows:
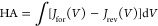
10where the integral is taken over the sweeping voltage range and *J*_for_ and *J*_rev_ represent current density in forward or reverse directions, respectively. Based on the sign of the obtained values of HA, hysteresis can be classified as positive or negative. We note that in the literature, depending on the current measured in forward and reverse sweeps at a given voltage, they are also termed inverted (*J*_for_ > *J*_rev_) or normal hysteresis (*J*_for_ < *J*_rev_). Both types can be observed in a given device.^47,48^

Along with the HA under the *J*–*V* curve, the shape of the curve provides information regarding the charge carrier transport. An ideal dark *J*–*V* curve should follow an exponential behavior. However, depending on the charge extraction dynamics in the device, S-shapes or N-shapes could appear. Unlike S-shapes, which originate from limited charge extraction at the transport layer interface,^49−51^ N-shapes have been attributed to tunneling barriers.^52,53^

*J*–*V* measurements were performed as a function of voltage sweep rates (200 mV/s to 20 kV/s) as well as temperature (Figure S9). The area between forward (0 to 1.2 V) and reverse (1.2 to 0 V) sweeps is calculated according to eq 10. A strong sweep rate and temperature dependence of *J*–*V* curves is observed in all devices. The sweep-rate-dependent measurements are fully reproducible with 30 s of settling time after forward and reverse sweeps.

**Figure 3:**
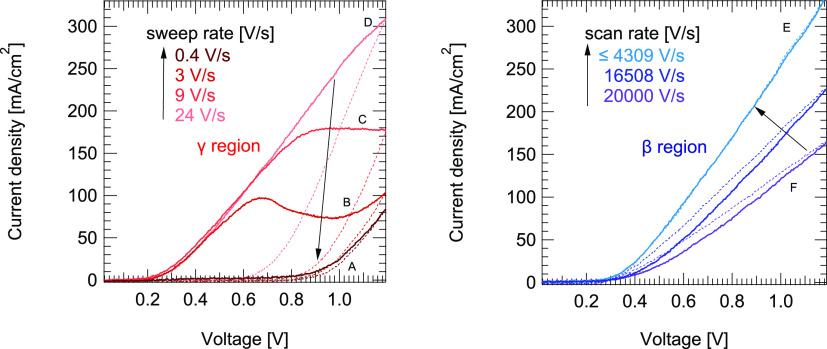
*J*–*V* sweeps of the triple-cation solar cell measured as a function of sweep rate at 360 K. High sweep rate sweeps show a steady increase in the exponential slopes (right), while measurements at low sweep rates lead to N-shaped curves in the forward direction with significant negative hysteresis (left). The reverse sweeps show no sign of N-shaped deformation and follow an exponential behavior. The hysteresis area as a function of frequency is labeled as A–F in Figure 4.

Similar to the IS, at high temperatures, two distinct regions are observed at low and high sweep rates, respectively. An example is shown in Figure 3. Initially, as we decrease the sweep rate, a steady increase in the exponential slopes of the *J*–*V* curves is seen. A further decrease in sweep rates leads to rather identical *J*–*V* curves until, at low sweep rates, *J*–*V* curves change drastically toward N-shapes. This intriguing behavior is dynamic and eventually retains exponential behavior as we further move to a slower sweep rate. Most importantly, this behavior is highly asymmetric and resembles tunnel diode characteristics in a reverse sweep. In contrast to a tunnel diode, here an N-shape occurs only in the forward sweeps. This pattern is consistently observed in all four configurations, regardless of the interfacial modifications.

As seen in Figure 3 (right), a less pronounced negative hysteresis area is observed at high sweep rates. In contrast, lower sweep rates (left) lead to significant positive hysteresis area.

### Correlation between IS, *J*–*V*, and HA

**Figure 4:**
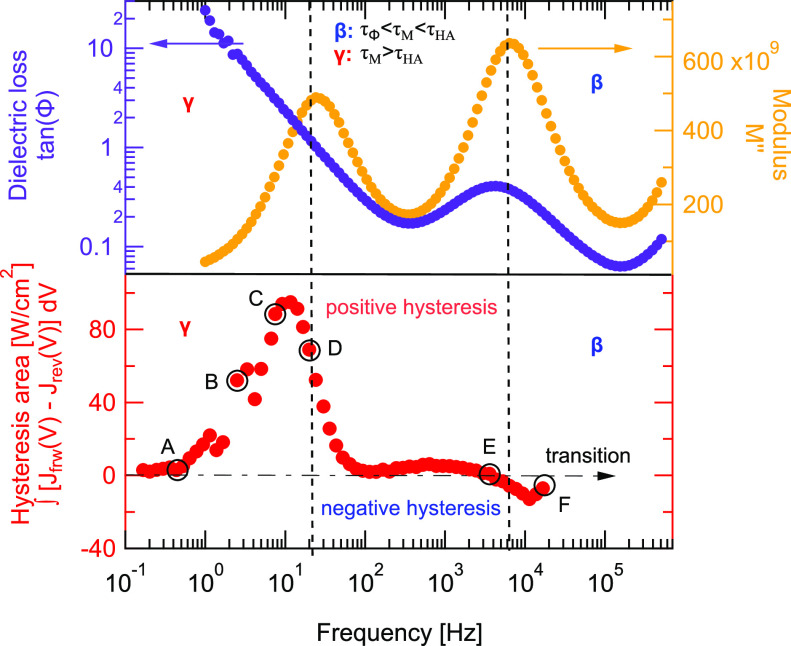
A comparison of IS (top), hysteresis area (bottom) as a function of frequency of triple-cation polycrystalline device measured at 360 K. The low- and high-frequency features of hysteresis area coincide with β and γ defects observed in IS. Both methods show comparable features. Therefore, negative hysteresis originates from β, while positive hysteresis is due to γ. The markers A–F indicate the corresponding *J*–*V* curves in Figure 3.

As IS probes both ionic and polarization effects, a correlation to *J*–*V* hysteresis helps to understand the hysteresis loops since output lagging the input in *J*–*V* curves is equivalent to the loss tangent tan(ϕ) extracted as a function of frequency. If the frequency of the *J*–*V* sweep is sufficiently high, the ionic defects cannot follow the applied voltage. At very low frequencies, ions move with an applied electric field as in steady-state conditions. Hence, both conditions lead to a negligible hysteresis area in *J*–*V* curves. At intermediate frequencies, ions do not reach a steady-state distribution, leading to a delayed current response. As this delay is highest at ω_c_, when the characteristic frequency and the applied voltage sweep frequency are at resonance, i.e., ω_c_ ≃ ω_sweep_, the maximum hysteresis area can be expected. A comparison of IS and HA as a function of the frequency of a polycrystalline triple-cation device with HTL-modification is shown in Figure 4. The corresponding *J*–*V* curves are shown in Figure 3. The low- and high-frequency features of the hysteresis area coincide with β and γ defects observed in IS. This clearly indicates that we are observing the same time constants as in IS, i.e., the same defects. The Arrhenius representation of migration rates obtained from IS and HA are summarized in Figure S7.

As can be seen in Figure 3 (left), N-shapes in *J*–*V* curves start to appear in the low-frequency part of the γ peak. The formation of the N-shapes has been attributed to a tunnel junction in MAPbI_3_ perovskite solar cells and is assigned to the HTL/MAPbI_3_ interface. A local heavy doping caused by electrostatic dipoles at a rough interface,^54^ ion accumulation induced doping resulting in reduced depletion width at the interface,^55^ slow rate of charge carrier capture, or release associated with structural reorientation of a molecule^56^ were proposed as the origin of transient tunnel junction.

Since we measured not only *J*–*V* but also IS, we have the possibility to combine both interpretations as both methods show the same time constants with comparable activation energies (Figure S7). A major contribution to the IS spectra at low frequencies is from γ. Toward low frequencies, the dielectric loss in Figure 4 (top), increases rapidly with constant slope, unlike at high frequencies where it is equal to ω = 1/τ_tan(ϕ)_ with a constant time delay. This observation suggests that in the γ region the ratio between the energy lost to energy stored in the system increases. This leads to a massive charge accumulation at the interface as we decrease the frequency. Since we did not observe a significant dielectric response at low frequencies in −ωd*C*/dω plots (Figure 1), a reorientation of molecules or formation of dipoles may not be a dominant origin for N-shapes in *J*–*V* curves. Hence, a charge carrier extraction barrier at the perovskite/transport layer interface formed by defect γ is likely the cause for the observation of N-shapes in our devices.

In the literature, positive and negative hysteresis in perovskites has been explained through charge extraction barriers associated with ionic defect accumulation at the interfaces^47,57−62^ or reversed band bending.^63^ These ionic defects may contain different origin with opposite polarity.^59^

IS probes ionic defect movement and is sensitive to the delay between the applied voltage and measured current, i.e., phase shift (ϕ). The sweep-rate-dependent *J*–*V* curves probe the transport of electronic charge carriers as a function of the same dynamic ion distribution. A phase shift associated with the ion distribution could also cause different hysteresis types in *J*–*V* curves. Therefore, different types of ionic conduction involved with β and γ could lead to the transition from negative to positive hysteresis. The former is due to ionic conductivity associated with dielectric relaxation, while in the latter case the conduction is restricted by the perovskite/transport layer interface. In order to verify if the defect signature arises from transport layers, a comparable transport layer stack without perovskite was measured, and no defect signature was observed.^64^ This proves that both β and γ defects originate from the perovskite layer.

At low frequencies, ionic defects move as under steady-state conditions in thermal equilibrium. As we increase the frequency, ionic defects start migrating through the device and accumulate at the perovskite-transport layer interface. Coulumb interaction stores the energy; ionic defects come closer to the transport layer than in steady-state conditions (Debye layers) which suggests a resonant movement. As can be seen in Figure 3 (left) and Figure 4 (bottom), N-shapes only start to appear on the low-frequency part of the γ peak. In terms of N-shapes, this oscillating movement of ionic defects can be seen as a potential well located at the perovskite/transport layer interface.^27^

### Formation of the N-Shaped Dynamic *J*–*V* Curves: A Scenario

At short-circuit conditions, ionic defects accumulate in the proximity of their corresponding transport layers:^10,65^ the negatively charged ions at the ETL or positively charged ions at the HTL. For the sake of simplicity, we focus on negatively charged ions.

During the voltage sweep, due to increasing applied voltage, the negatively charged ionic defects start at the perovskite/ETL interface and accelerate toward the HTL. If the ionic defect distribution arrives at the opposite transport layer before it broadens due to drift and diffusion, ionic defects reach the HTL. By this effect the ionic defect distribution width might decrease compared to the steady-state Debye distribution. If the ion density is large enough, a significant electric field will be created by the ionic defects in the opposite direction to the applied electric field. This acts as an extraction barrier for electrons and leads to a decrease in the local electric field. In our scenario, this creates the N-shape in the *J*–*V* curve. Further, this space charge will redistribute into a Debye layer with increasing time at a given voltage, leading to the *J*–*V* curve retaining exponential behavior at higher voltages.

During the reverse sweep, the same process will occur with the difference that the negative ions now accelerate from the positively charged HTL to the negatively charged ETL. However, the space charge region around the ETL is dominated by injected electrons with an electron charge carrier density exceeding the ion density. Since the electric field is generated by a combination of all charges located in that region, according to the Poisson equation, this does not lead to a significant electric field contribution of the ions, and no N-shapes are observed.

At even lower sweep rates or frequencies, the ionic defect distribution broadens before it reaches the transport layer and directly forms a Debye layer. An exponential behavior is observed.

### The Defect Polarity of γ

In order to identify the ionic defect polarity and to understand which transport layer is causing the N-shapes, we looked at a series of modified ETL or HTL layers. Modulus and conductivity plots can be seen in the Figure S5. The peak heights of β and γ do not change in the modulus plot, in contrast to the low-frequency limit of conductivity. Since *M*″ ∝ 1/*C*, as defect densities scale with capacitance peak height,^25^ this implies that the modification may not change the defect densities in the perovskite. However, according to the Nernst–Einstein relation, the modification influences the mobility of the ionic defects.^25^ The HTL-modification leads to faster movement of ionic defects, while the ETL or dual-modification reduces their diffusion. Hence, we observe a smaller hysteresis area in the case of ETL and dual-modified devices, while a higher hysteresis area is observed in reference and HTL-modified devices.

**Figure 5:**
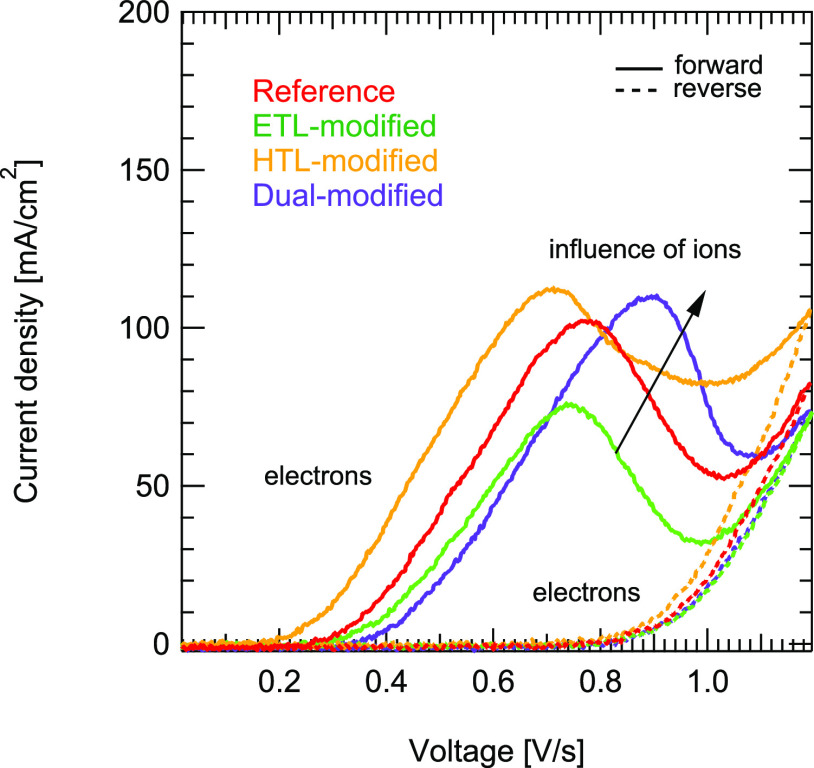
Comparison of N-shapes observed in *J*–*V* curves as function interface modifications measured at 4 V/s at 360 K.

A modification of the HTL leads to better charge extraction at the HTL.^36^ As the same modification layer is applied at the ETL, it could be considered that this will decrease the electron density at the ETL. This can be seen in Figure 5; at lower voltages, current density is higher for the HTL-modified device compared to all other devices. The HTL-modified device shows significantly less current drop in the N-shape regime compared to the rest, implying that the electric field drop associated with space charge at the HTL is reduced. Since we observe the same capacitance peak heights, i.e., same amount of ionic defects in all devices, this implies that due to the HTL-modification, the electric field created by the ionic defects is reduced compared to all other devices. In terms of the proposed model this can be seen as negatively charged ionic defects moving toward an increased positive space charge around the HTL. For the dual-modified device, it is likely that by modifying both ETL and HTL the recombination mechanisms of electrons and holes are influenced, which makes the assignment of low ionic defect densities difficult.

This suggests that the perovskite/HTL interface is responsible for formation of the N-shape in *J*–*V* curves, which is consistent with the literature.^55,66^

### Origin of β and γ Defects

In all polycrystalline triple-cation devices, the imaginary part of the modulus (*M*″) shows similar peak heights for both β and γ (Figure S4). Since *M*″ ∝ 1/*C*, we observe a similar capacitance contribution from β and γ. Since ion migration is associated with long-range conduction, this might suggest that we are observing the same species migrating. In the case of γ, the migration is limited by the perovskite/transport layer interface, while β is associated with dielectric relaxation. As ionic defects always form in pairs, though β and γ appear to have the same ionic origin, they may not contain the same polarity. In principle, deep level transient spectroscopy (DLTS) can identify the defect polarity if the effective doping type of the material is known. In our measurements of DLTS, β and γ always appeared as opposite polarity.^37^ As an example, the DLTS spectrum of a single-crystal device measured at 300 K is shown in Figure S6.

However, due to the Coulomb interaction of neighboring defects, the effective electric field at an ionic defect site will shift in both magnitude and phase from the external applied field.^27,28^ Hence, as the ionic defects migrate, they experience more lag from their neighbors, leading to a transition from capacitive to inductive behavior.^27,28^ This can be seen in Figure 1, the regime below γ peak position (below 1 Hz) in IS starts to show an inductive behavior with increasing resistance as the frequency rises, while the rest of the spectra resembles a capacitive behavior. In the case of enhanced conductivity, such as at higher temperatures, external applied bias, or higher illumination intensity this inductive behavior can be seen as a clear negative loop in Nyquist plots^66,67^ within the measurement frequency range. This inductive behavior is an intrinsic property of the perovskite and observed in both n-i-p^68^ and p-i-n^17^ device structure, without transport layers,^69^ and also in single-crystal MAPbBr_3_ devices.^70^

As DLTS only shows a change in polarity at the γ region^24^ (Figure S5), it might be possible that the capacitive/inductive phase change is detected as a defect with opposite polarity. Moreover, the sign convention of capacitance transience in DLTS depends on whether the perovskite is a p-type or n-type semiconductor. Hence, it is still debatable as to how far the charge separation applies to the investigated device geometry. Therefore, there is a possibility that β and γ are caused by same ionic species, even the same charge type. However, further theoretical and experimental work is required to confirm their polarity.

## Conclusion

We investigated ionic conduction in perovskite solar cells by a combination of impedance spectroscopy and sweep-rate-dependent *J*–*V* measurements. A joint representation of modulus, permittivity, impedance, and conductivity formalism is valuable for identifying not only different types of conduction mechanisms but also the defect location. We observe that there are two defects, β and γ. Both are bulk defects that originate from long-range ionic conduction. The defect β is associated with dielectric relaxation and is likely an iodine-related defect.

Depending on the resistive and capacitive contributions of the defects to the impedance and modulus spectra, we assigned γ to ionic conduction limited by an interface. By comparing single-crystal MAPbI_3_ and polycrystalline triple-cation devices, based on the N-shapes observed in *J*–*V* curves at lower sweep rates of the polycrystalline triple-cation device, we concluded that conduction of γ is limited by the perovskite/transport layer interface.

Both IS and hysteresis area between sweep-rate-dependent *J*–*V* curves lead to same time constants, i.e., same defects. The conduction types associated with β and γ are the causes of positive and negative hysteresis in *J*–*V* curves. We proposed that the tunnel diode like characteristics that we call N-shapes in *J*–*V* curves are due to accumulation and redistribution of ions in the perovskite close to the hole transport layer.

Based on sweep-rate-dependent *J*–*V* curves performed on a series of devices with interfacial modifications, we showed a possibility of breaking the symmetry in the device and further assigned γ to an ionic defect of negative polarity such as an iodide interstitial. There is a possibility that both defects β and γ originate from the same ionic species, even the same polarity. Our observations through combined interpretation of IS and *J*–*V* curves provide clear evidence of ionic conduction and its correlation to hysteresis in perovskites.

## Methods

### Defect Spectroscopy

All devices were measured using a setup consisting of a Zurich Instruments MFLI lock-in amplifier with MF-IA and MF-MD options, a Janis ST500 cryo probe station with a Lakeshore 336 temperature controller. We performed impedance spectroscopy measurements under dark and at short-circuit conditions, in the temperature range of 240 K to 360 K in 20 K steps, using liquid nitrogen for cooling. An AC voltage with an amplitude of 20 mV was applied over a wide range of frequencies (0.6 Hz < ω < 3.2 MHz). DLTS measurements were performed at 300 K, by applying an AC frequency of 100 kHz with amplitude of 20 mV along with a bias from 0 to 1 V for 100 ms. The transients were measured over 30 s and averaged over 30 single measurements.

### Sweep-Rate-Dependent Hysteresis

Dark *J*–*V* curves were obtained by applying a triangular pulse starting from 0 to 1.2 V (and to back) to the device, and the respective current response was monitored through an oscilloscope (Lecroy waverunner 610 Zi). Since the oscilloscope can only measure voltage signal, a transimpedance amplifier DHPCA-100 was used to convert current to voltage. A waveform generator Keysight 33600A was used as the voltage source, and the frequency of the pulse was varied. An HF-amplifier in a series connection was used in order to reduce the output resistance of the function generator to 1 Ohm. A settling time of 30 s was considered in between each measurement.

### MAPbI_3_ Single-Crystal Device Fabrication

#### Materials

Methylammonium Iodide (MAI) was purchased from Greatcell Solar Limited (Australia). Lead(II) iodide (PbI_2_, 10-mesh beads, ultradry, 99.999%) was purchased from Alfa Aesar. γ-Butyrolactone (GBL, > 99%) and toluene (anhydrous, 99.8%) were purchased from Sigma-Aldrich. Polytriarylamine (PTAA, > 99%, sublimed, Mw: 17 k) was purchased from Xi’an Polymer Light Technology Corp. All materials were used as received without any further purification.

#### Substrate Preparation

The 5 cm × 5 cm ITO substrates (5 cm × 5 cm) were sonicated in soap, DI water, acetone, and IPA sequentially, followed by an ultraviolet-ozone surface treatment for 15 min. PTAA solution was spin-coated onto substrates for 30 s at 4000 rpm, and the substrates were subsequently annealed at 100 °C for 10 min.

#### Growth and Fabrication of Single-Crystal Perovskite Solar Cells

Single-crystal solar cells were grown and fabricated following the reported works.^71^ A 1.55 M solution of MAPbI_3_ in γ-butyrolactone (GBL) was prepared by dissolving equimolar amounts of MAI and PbI_2_ in GBL at 60 °C by stirring for a few hours. A 60 μL sample of the solution was placed on a PTAA-coated substrate preheated at 60 °C on the hot plate and enclosed by another PTAA-coated substrate. The temperature was then increased gradually to 120 °C to induce nucleation and growth. The substrates were subsequently separated using a blade and were cooled slowly to room temperature. Crystal sizes ranging from 1 to 3 mm^2^ were obtained. C_60_ (20 nm) and BCP (3 nm) were thermally evaporated at a rate of 0.1 Å/s to form an electron transport layer. Polyimide tape (Kapton) was used to cover the edges of single crystals to prevent short-circuiting between the top electrode and ITO. Finally, Cu (80 nm) was evaporated at a rate of 1 Å/s to complete the devices. Before photovoltaic measurement, a photomask was properly placed on the glass side of the substrate for each device.

### Fabrication of Triple-Cation Devices with Interface Modifications

Prepatterned ITO/glass substrates were sequentially cleaned with acetone and 2-propanol (IPA) by ultrasonication for 15 min in each solvent. The ITO/glass substrates were then dried with N_2_ and treated with oxygen plasma at 100 mW for 10 min. The HTL and the perovskite films were fabricated in a drybox (relative humidity <2%), while the ETL and the contacts were deposited inside a glovebox filled with inert atmosphere N_2_. For reference devices, an HTL of 10 nm thickness made of PTAA with a concentration of 1.5 mg dissolved in toluene was spin-coated at a speed of 2000 rpm for 40 s, using 30 μL of solution, and then annealed at 100 °C for 10 min. After the annealing step, the samples were washed by 50 μL of DMF by spin-coating it on the prepared PTAA films dynamically, 5 s into a 30 s spinning step at 4000 rpm.

The perovskite precursor solution (1.2 M) contained mixed cations (Pb, Cs, FA, and MA) and halides (I and Br) dissolved in a solvent mixture (DMF/DMSO = 4/1) according to the formula Cs_0.05_(FA_0.83_MA_0.17_)_0.95_Pb (I_0.9_ Br_0.1_)_3_ with an excess of PbI_2_ of 1%. The perovskite layer was deposited via a two-step spin-coating procedure with 1000 rpm for 12 s and 5000 rpm for 27 s. Before spinning, 40 μL of perovskite precursor solution was applied to the sample statically and a mixture of antisolvents (chlorobenzene (CB)/IPA = 9/1, 150 μL) was dripped on the spinning substrate, 21 s into the second spin-coating step. Subsequently, the samples were annealed at 100 °C for 30 min. The ETL was dynamically deposited from a PC_61_BM solution (20 mg/mL in CB) and spin-coated onto the perovskite layer dynamically, by pipetting 20 μL of solution, 5 s into a spinning step at the speed of 2000 rpm for 30 s (with a ramping speed of 1000 rpm/s) and annealed for 10 min at 100 °C. Next, thin layers of BCP (0.5 mg/mL in IPA) were spin-coated dynamically, by pipetting 40 μL of solution, 5 s into a spinning step at 4000 rpm for 30 s (with a ramping rate of 1000 rpm/s) as hole blocking layers. The small-area devices with an area of 4.5 mm^2^ were completed by thermal evaporation of Ag (80 nm). The devices with modified interfaces were prepared by dissolving a small amount of the PEAI-cations in DMF (20 mM) used for washing the PTAA and in the mixture CB/IPA (0.5 mM) used in the antisolvent step. All other device fabrication steps were unchanged.

## Supporting Information Available

Graphical illustration of long-range and short-range conduction (Figure S1), conductivity as a function of frequency (Figures S2, S3, and S5), hysteresis area (sweep rate, temperature, interface modification) (Figure S4), DLTS of single-crystal device (Figure S6), Arrhenius representation (Figure S7), and IS and IV (temperature, interface modification) (Figures S8–S11) (PDF)
