# Supplementary material for: Hysteresis and Its Correlation to Ionic Defects in Perovskite Solar Cells
Source: J Phys Chem Lett. 2024 Jan 29;15(5):1363–72. doi: 10.1021/acs.jpclett.3c03146 (PMC10860142; doi:10.1021/acs.jpclett.3c03146)
Supplement: Supplementary file 1 — jz3c03146_si_001.pdf [file jz3c03146_si_001.pdf]

Supplementary Information: Hysteresis and its Correlation to Ionic Defects in Perovskite Solar Cells

Sandhya Tammireddy,[†] Muhammad N. Lintangpradipto,[‡] Oscar Telschow,[¶] Moritz H. Futscher,[∥] Bruno Ehrler,[⊥] Osman M. Bakr,[‡] Yana Vaynzof,[¶] and Carsten Deibel[*,†]

†*Institut für Physik, Technische Universität Chemnitz, 09126 Chemnitz, Germany*

‡*KAUST Catalysis Center (KCC), Division of Physical Sciences and Engineering (PSE), King Abdullah University of Science and Technology, Thuwal 23955-6900, Kingdom of Saudi Arabia*

¶*Chair for Emerging Electronic Technologies, Technical University of Dresden, Nöthnitzer Str. 61, 01187 Dresden, Germany*

§*Leibniz-Institute for Solid State and Materials Research Dresden, Helmholtzstraße 20, 01069 Dresden, Germany*

∥*Laboratory for Thin Films and Photovoltaics, Empa - Swiss Federal Laboratories for Materials Science and Technology, 8600 Dübendorf, Switzerland*

⊥*Center for Nanophotonics, AMOLF, Science Park 104, 1098 XG Amsterdam, The Netherlands*

E-mail: deibel@physik.tu-chemnitz.de

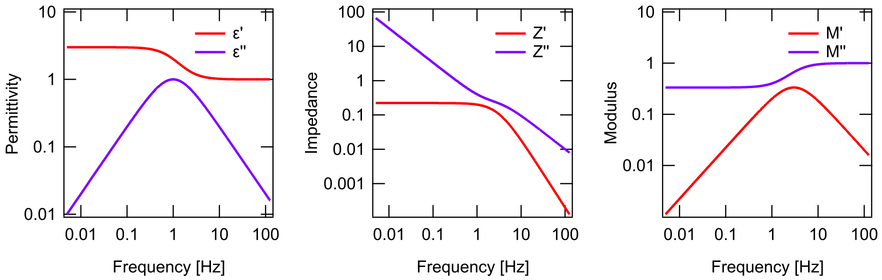

(a) Short-range conduction

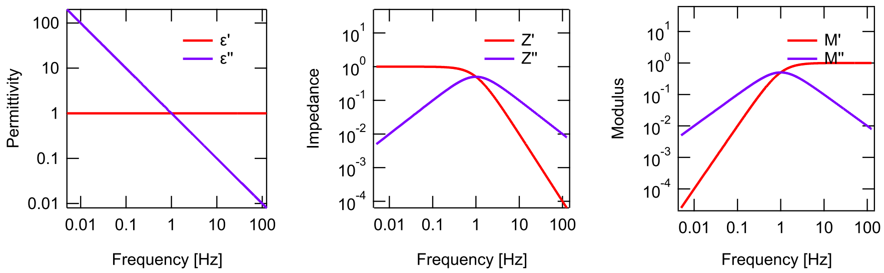

(b) Long-range conduction

Figure S1: Graphical illustration of identifying short-range (a) and long-range conduction (b) via permittivity, impedance, modulus representations. A loss peak in the permittivity ε'' and different relaxation times in impedance and modulus i.e., $\tau_Z \neq \tau_M$ is an indication for short-range conduction, while absence of loss peak with equal relaxation times of impedance and modulus $\tau_Z = \tau_M$ is regarded as long-range conduction.

Analysis of conductivity spectrum

The frequency dependent conductivity $\sigma(\omega, T)$ spectrum analysed by the Jonscher universal power law for disordered solids

$$\sigma(\omega, \mathrm{T}) = \sigma_{\mathrm{lf}}(\mathrm{T}) + \omega\varepsilon_0\bar{\varepsilon}'' + \mathrm{A}(\mathrm{T})\omega^{\mathrm{s}} \tag{S1}$$

where, $\bar{\varepsilon}''$ is the imaginary part of the permittivity associated with dielectric relaxation. Ideally, the dielectric relaxation can be expressed by Debye function, which assumes that all relaxation process in the system decay with same relaxation time. However, in contrast to ideal Debye relaxation, the measured loss peaks are asymmetrically broadened and the relaxation steps are spread out. In disordered solids, this phenomena is attributed to a non-exponential decay associated with

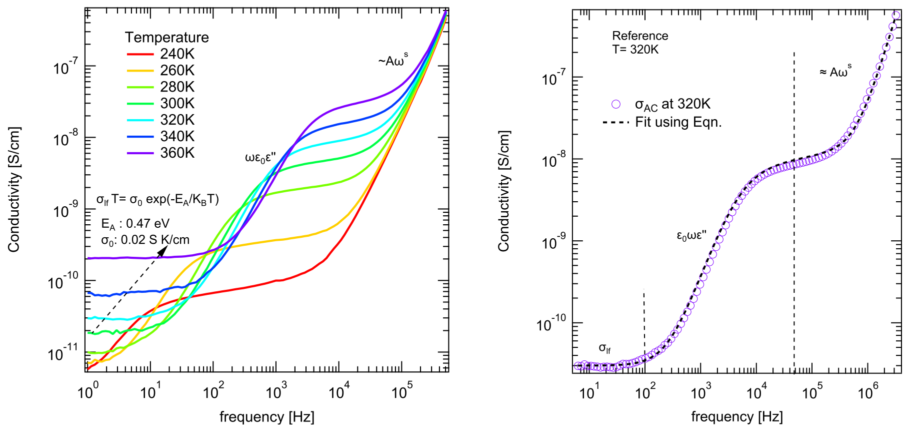

Figure S2: Temperature dependent conductivity as function of frequency (left) and an example of fitting procedure according to Eqn (6). Three distinct regions at low, middle and high frequencies are highlighted. The additional dielectric response is fitted by using Havrilliak-Negami function.

distribution of relaxation times. Mathematically, the non-exponential decay can be accounted by Havrlliak-Negami function,[S1] which can be expressed as,

$$\bar{\varepsilon}''(\omega) = \Delta\varepsilon \, \frac{\sin\theta}{\left[1+(2\omega\tau)^{\alpha}\ \cos(\frac{\alpha\pi}{2})+(\omega\tau)^{2\alpha}\right]^{\frac{\bar{\beta}}{2}}}, \quad \text{where} \quad \theta = \bar{\beta}\arctan\frac{(\omega\tau)^{\alpha}\sin(\frac{\alpha\tau}{2})}{1+(\omega\tau)^{\alpha}\cos(\frac{\alpha\pi}{2})} \qquad \text{(S2)}$$

where $\Delta\varepsilon = \varepsilon_s - \varepsilon_\infty$ is the dielectric strength of relaxation process, τ is the relaxation time, α and $\bar{\beta}$ are the broadening parameters vary in the range $0 \leq \alpha < 1$ and $0 < \bar{\beta} \leq 1$. For the fit shown in Figure.2, $\alpha = 0.9$, $\beta = 1$, which indicates the symmetrical broadening of distribution of relaxation times in the measured device.

References

[S1] Floudas, G. In *Polymer Science: A Comprehensive Reference*; Matyjaszewski, K., Möller, M. B. T. P. S. A. C. R., Eds.; Elsevier: Amsterdam, 2012; pp 825–845.

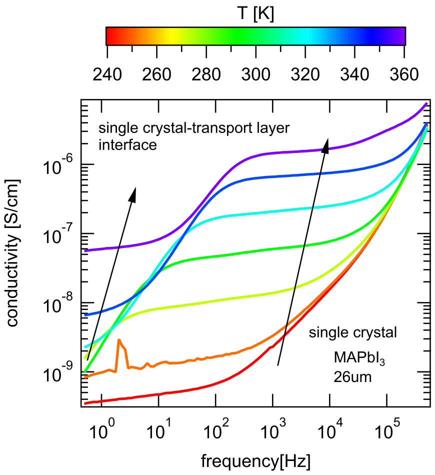

Figure S3: Frequency dependent conductivity of single crystal $MAPbI_3$. Since there are no grain boundaries, low frequency feature attributed to perovskite/transport layer interface while high frequencies represent bulk response.

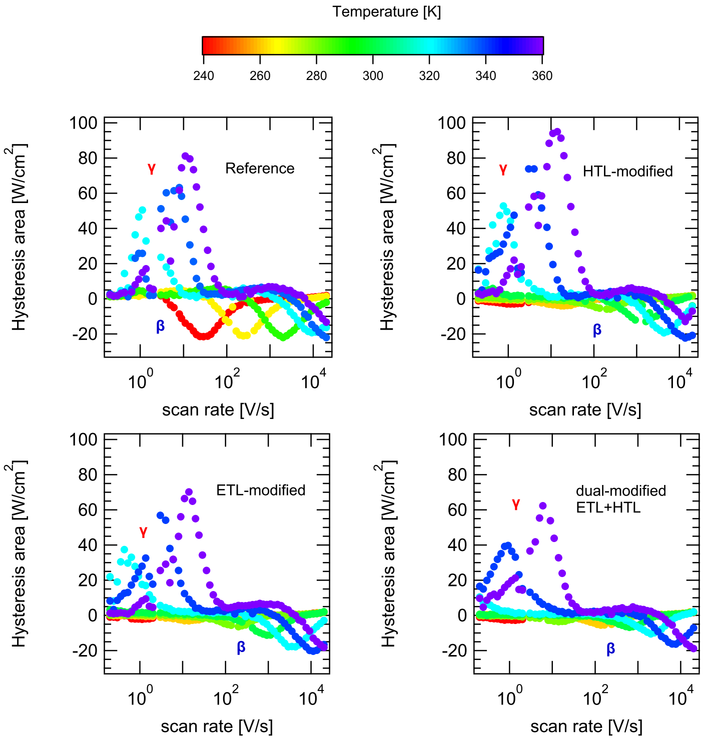

Figure S4: Hysteresis area as function of scan rate and temperature of devices with interface modifications. Both positive and negative hysteresis observed in all devices. Negative hysteresis is prominent in reference device compared to others. Positive hysteresis of ETL-modified and Dual-modified devices are less prominent while HTL-modified device show higher values compared to reference device.

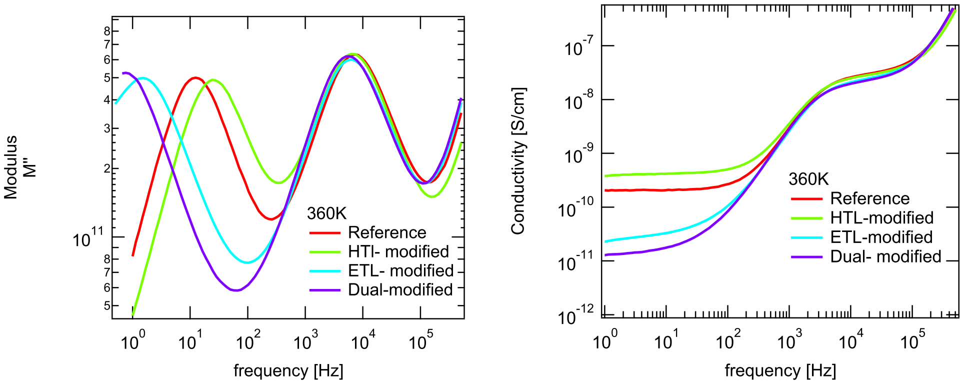

Figure S5: Modulus and conductivity comparison of devices as function of interface modification. As modulus is proportional to 1/C, since the peak heights of γ and β are same, the interface modification does not influence the defect density of perovskite but changes the low frequency approximation of conductivity, σ_{lf} by influencing the diffusion of ions moving towards the perovskite/transport layer interface.

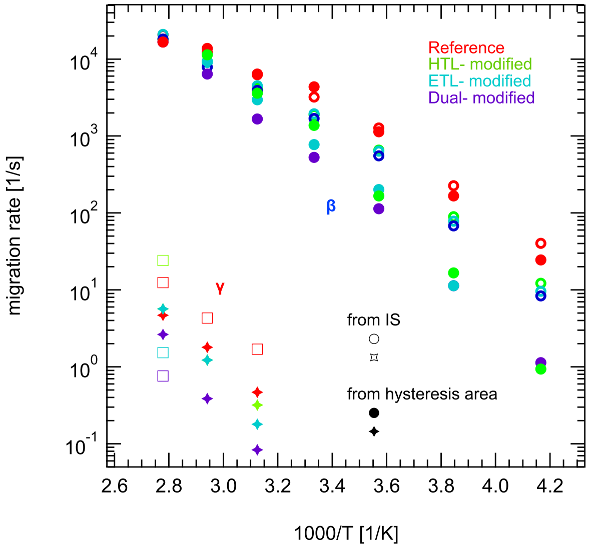

Figure S6: A comparison of migration rates obtained from IS and hysteresis area as function of temperature is shown. The defect γ from IS is obtained from imaginary part of modulus M''.

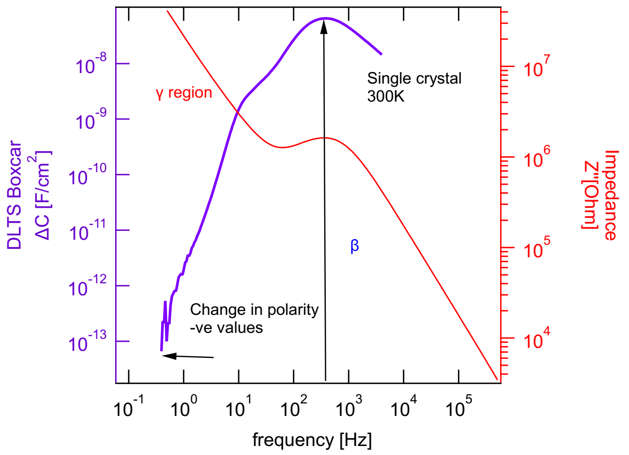

Figure S7: A comparison of impedance (Z'') and DLTS-Boxcar plot of single crystal device measured at 300K is shown. Positive and negative peaks in DLTS-Boxcar signal are considered as signs for defects with different polarity. The defect β can be seen as distinct peak with positive values while, the frequencies below γ region show negative values. This suggests β and γ are of apposite polarity. However, a phase shift associated with capacitive/inductive behaviour may also change the capacitance sign. Hence, there is a possibility that β and γ are of same ionic defect origin.

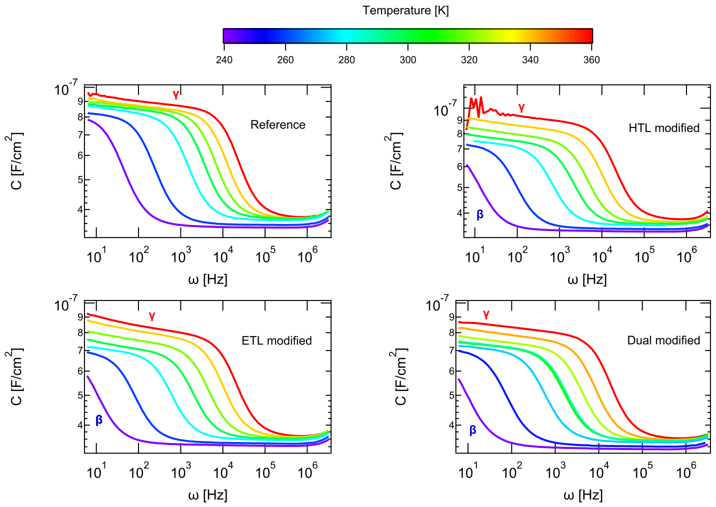

Figure S8: Capacitance-frequency spectra as function of temperature of devices with surface modifications. A low frequency, high temperature response γ and a high frequency response γ are highlighted.

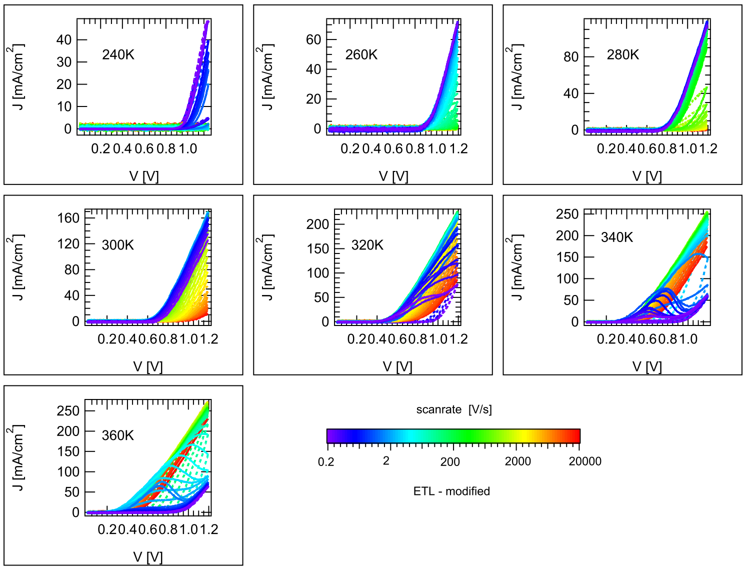

Figure S9: Scan rate and temperature dependent J–V of ETL-modified device is shown. Below room temperatures show an exponential behaviour, while high temperatures show N-shape towards low scan rates.

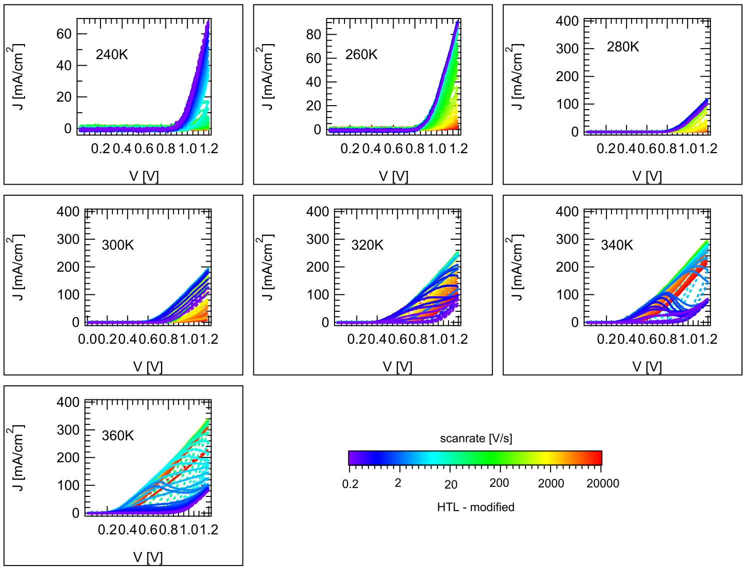

Figure S10: Scan rate and temperature dependent J–V of HTL-modified device is shown.

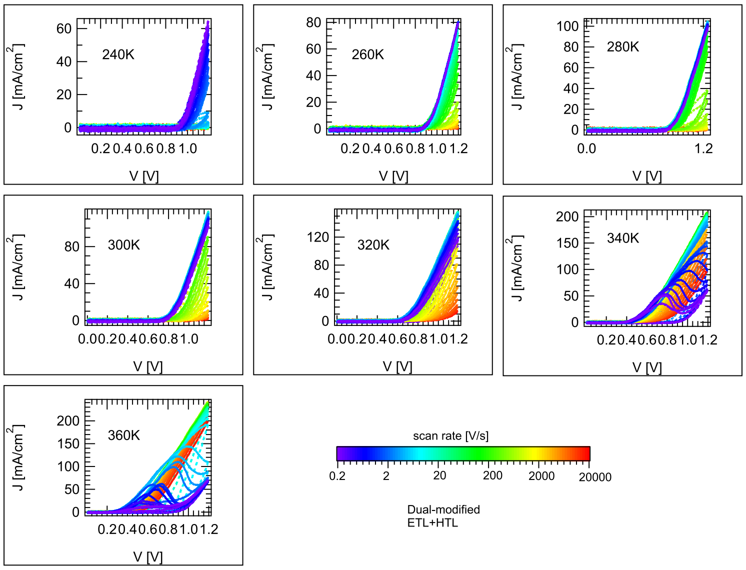

Figure S11: Scan rate and temperature dependent J–V of Dual-modified device is shown.
